# Prenatal Diagnosis of Bilateral Ectrodactyly and Radial Agenesis Associated with Trisomy 10 Mosaicism

**DOI:** 10.1155/2013/592702

**Published:** 2013-01-13

**Authors:** Jonathan Lévy, Jean-Marie Jouannic, Julien Saada, Ferdinand Dhombres, Jean-Pierre Siffroi, Marie-France Portnoï

**Affiliations:** ^1^Service de Génétique et d'Embryologie Médicales, Hôpital Armand Trousseau, APHP, UPMC, 75012 Paris, France; ^2^Centre Pluridisciplinaire de Diagnostic Prénatal de l'Est Parisien, Pôle de Périnatalité, Hôpital Armand Trousseau, APHP, UPMC, 75012 Paris, France

## Abstract

Ectrodactyly or split hand and foot malformations (SHFMs) are rare malformations of the limbs, characterized by median clefts of the hands and feet, syndactyly, and aplasia and/or hypoplasia of the phalanges. They represent a clinically and genetically heterogeneous disorder, with both sporadic and familial cases. Most of the genomic rearrangements identified to date in some forms of SHFM are autosomal dominant traits, involving various chromosome regions. Bilateral radial ray defects comprise also a large heterogenous group of disorders, including trisomy 18, Fanconi anemia, and thrombocytopenia-absent-radius syndrome, not commonly associated with ectrodactyly. The present paper describes a case of ectrodactyly associated with bilateral radial ray defects, diagnosed in the first trimester of pregnancy, in a fetus affected by trisomy 10. Only four cases of sporadic and isolated ectrodactyly, diagnosed by ultrasonography between 14 and 22 weeks' gestation, have been reported. To our knowledge, the present case is the first report of mosaic trisomy 10 associated with SHFM and radial aplasia. Trisomy 10 is a rare lethal chromosomal abnormality, most frequently found in abortion products. Only six liveborn mosaic trisomy 10 infants, with severe malformations, dead in early infancy, have been reported. A severe clinical syndrome can be defined, comprising ear abnormalities, cleft lip/palate, malformations of eyes, heart, and kidneys, and deformity of hands and feet and most often associated with death neonatally or in early infancy.

## 1. Introduction

Ectrodactyly or split hand and foot malformations (SHFMs) (OMIM 183600) are rare malformations of the limbs, characterized by median clefts of the hands and feet, syndactyly, and aplasia and/or hypoplasia of the phalanges. They represent a clinically and genetically heterogeneous disorder, with both sporadic and familial cases. The malformation can be isolated, typically inherited in an autosomal dominant pattern with variable expression, or as part of a syndrome, as in the ectrodactyly-ectodermal dysplasia-clefting (EEC) syndrome (OMIM 225280), all showing variable expressivity and reduced penetrance. Bilateral radial ray defects comprise also a large heterogenous group of disorders, including trisomy 18, Fanconi anemia, and thrombocytopenia-absent-radius (TAR) syndrome, not commonly associated with ectrodactyly [1].

The present paper describes a case of ectrodactyly associated with bilateral radial ray defects, detected prenatally using ultrasonography, in a fetus affected by trisomy 10. 

## 2. Clinical Report

**Figure 1:**
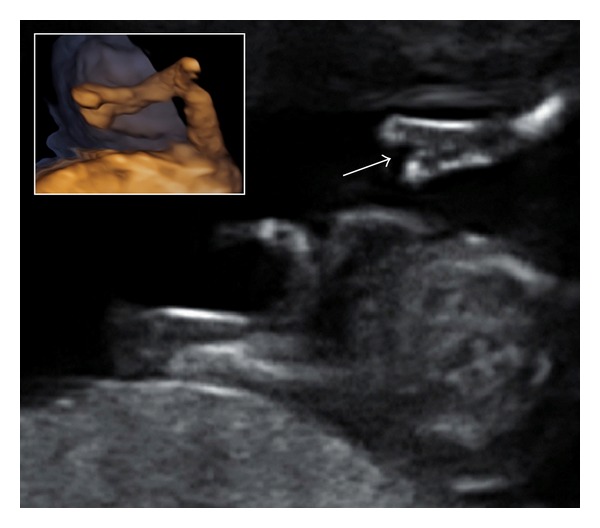
Ultrasound examination at 11 + 5 weeks' gestation: cleft hand (arrow) and three-dimensional view of the upper limb with associated radial agenesis.

A 31-year-old primigravid woman was referred to our center for a selective fetal reduction (FR), in a triplet pregnancy achieved by ovarian stimulation with gonadotropins because of an unexplained female infertility. The couple elected to proceed with FR because of a high risk for preterm birth and/or prenatal complications. Both parents were healthy. Ultrasound examination at 10 weeks' gestation showed a trichorionic triamniotic triplet pregnancy and revealed an increased nuchal translucency (NT) of 2.7 mm with a 54.8 mm crown-rump length in one fetus. This foetus was also presenting with bilateral ectrodactyly, including median cleft of both hands with oligodactyly, and bilateral radial agenesis (Figure 1). No other abnormality was found. The two other fetuses were normal, with biometry consistent with gestational age. Neither the patient nor her husband had any bone or other anomaly, and there was no history of consanguinity or family history of malformations. At 11 + 5 weeks' gestation, the abnormal fetus was selected for FR, and a sample of amniotic fluid was collected by amniocentesis for cytogenetic analysis. At present, the twin pregnancy is ongoing, currently in its 6th month, and uneventful.

Standard chromosomes and FISH analysis on amniotic fluid revealed a mosaic male karyotype 47,XY,+10/46,XY, with trisomy 10 in approximately 80% of 50 metaphase cells and 300 nuclei analysed, in the abnormal fetus.

## 3. Discussion

Prenatal diagnosis of SHFM has previously been reported in fetuses either with a familial history of ectrodactyly or in association with the EEC syndrome [6–8]. Only four cases of sporadic and isolated ectrodactyly, diagnosed by ultrasonography between 14 and 22 weeks' gestation, have been reported [6, 9, 10]. One fetus presented with increased nuchal fold and abnormal hands with oligodactyly. The pregnancy was terminated. In a second case, of dichorionic twin pregnancy, ultrasonography at 19 weeks' gestation showed one normal twin and a second fetus with abnormal upper limbs including bilateral split hand and short forearms [6]. In all cases, amniocentesis showed a normal karyotype. 

Most of the genomic rearrangements identified to date in some forms of SHFM are autosomal dominant traits, involving various chromosome regions. Six SHFM loci have been identified, at 7q21 (SHFM1), 10q24 (SHFM3), 3q27 (SHFM4) resulting from mutations in the P63 gene, 2q31 (SHFM5), 12q13 (SHFM6), and Xq26 (SHFM2). SHFM3 abnormalities appear to represent a major cause of SHFM and are associated with submicroscopic tandem duplications (500–650 Kb) on chromosome 10q24 [11, 12]. The smallest duplicated region (325 kb) contained a disrupted extra copy of the dactylin gene (FBXW) and the entire LBX1, BTRC, POLL, and DPCD genes. However, there is no explanation how the observed 10q24 genomic rearrangement causes the phenotype of SHFM [12]. There is no obvious correlation between the size of the 10q24 duplication and the SHFM phenotype [12]. The affected individuals with larger duplications of chromosome 10q, including the SHFM3 locus, have a more severe clinical phenotype including mental retardation. The hand/foot anomalies in the patients are generally mild and do not always fall in the SHFM spectrum. 

In a cohort of patients with unexplained syndromic limb defects, analyzed by a-CGH, a 500 kb 10q24 microduplication was detected in two brothers with distal limb deficiencies associated with micrognathia, deafness, and renal hypoplasia. The more severely affected older brother had severe distal limb deficiencies including absent radii, and mental retardation. Limb malformations included deformation and bowing of distal radii in the younger [12]. SHFM cases at the SHFM3 locus seem to have an increased frequency of preaxial abnormalities and absent radial ray, as observed in our case with mosaic trisomy 10.

To our knowledge, the present case is the first report of mosaic trisomy 10 associated with SHFM and radial aplasia. Trisomy 10 is a rare lethal chromosomal abnormality, most frequently found in abortion products. Only six liveborn mosaic trisomy 10 infants, with severe malformations, dead in early infancy, have been reported [13]. A severe clinical syndrome can be defined, comprising ear abnormalities, cleft lip/palate, malformations of eyes, heart, and kidneys, and deformity of hands and feet and most often associated with death neonatally or in early infancy [13].

Prenatal diagnosis of trisomy 10 has been reported in only five cases, including ours [2–5] (Table 1). In all cases, increased nuchal translucency thickness or hygroma colli associated with limb defects were present. Detailed ultrasound examination showed additional features including micrognathia, facial cleft, limb abnormalities, cardiac defects, unilateral renal agenesis, and early intrauterine growth retardation. Termination of pregnancy was performed for all cases except one with IUD at 35 weeks' gestation. Postmortem examination showed a male growth-retarded fetus with a deep facial cleft, retromicrognathia, low-set ears, and short neck. Abnormal features also comprised hypoplastic third finger on the right hand, syndactyly of the right toes, and absence of toes on left foot. Renal and cardiac defects, imperforate anus, and agenesis of the corpus callosum were also observed [5]. In our case, diagnosed in the first trimester of pregnancy, the fetus presented with increased NT, ectrodactyly, and radial aplasia. No other malformation was detected before fetal reduction. 

Our case is the earliest prenatal diagnosis of SHFM, diagnosed by ultrasound examination at 10 weeks' gestation and the first paper of ectrodactyly and radial agenesis associated with trisomy 10. 

Diagnosis of trisomy 10 in the malformed fetus constituted help for genetic counseling to the parents and for the management of subsequent pregnancies. This result reinforces the recommendation to perform cytogenetic analysis when sporadic SHFM findings are detected during pregnancy. 

## Figures and Tables

**Table 1:** Clinical and cytogenetic findings in prenatal diagnosis of trisomy 10.

	Farrell et al. (1994) [2]	Schw$\ddot{\text{a}}$rzler et al. (1999) [3]	Knoblauch et al. (1999) [4]	Brizot et al. (2001) [5]	Present case
Maternal age	32	32	36	18	31
Gender	F	M	M	M	M
Gestational age of diagnosis (weeks)	15	12	13	12	10
Increased nuchal translucency	+	4.7 mm		5.4 mm	2.7 mm
Hygroma colli			+		
IUGR	+		+	+	
Generalized oedema			+	+	
Facial dysmorphism	+	+	+	+	
Prominent forehead		+	+		
Low set ears	+	+	+	+	
Micrognathia/retrognathia	+	+	+	+	
Cleft lip/palate	+	+	−	+	
Limbs defects	+	+	+	+	+
Radial agenesis					Bilateral
Deformity of hands/fingers	Duplicated right thumb	Left hypoplastic clenched V finger		Hypoplastic 3rd finger	Bilateral hand ectrodactyly
Deformity of feet/toes	Right 2/3 syndactyly of toes Hypoplastic left toes	Bilateral talipes	Proximally placed left first toe	Syndactyly 2/3/4 right toes Agenesis left toes Rockerbottom feet	
	Bilateral talipes Rockerbottom feet			Bilateral talipes	
Cardiac malformations		Atresia of tricuspid valve Hypoplastic right ventricule ASD	−	DORV VSD	
Renal agenesis	Right kidney	Right kidney	−	Left kidney	−
Other	Right lung agenesis	Diaphragmatic hernia	Liver agenesis	Microcephaly Corpus callosum agenesis Hypoplastic penis Imperforate anus	
	Intestinal malrotation				
Cytogenetic analysis					
Tissues/% trisomic cells	Amniocytes/100% Fetal Skin/100%	CVS/100%	CVS/95% Fetal skin/38%	CVS/100% Fetal blood/100%	Amniocytes/80%
Outcome					
Pregnancy termination	+	13 + 3 weeks	15 weeks		11 + 3 weeks (fetal reduction)
Stillborn				35 + 4 weeks	

(+) Present; (−) absent.

ASD: atrial septal defect; DORV: double outlet right ventricule; IUGR: intrauterine growth retardation; VSD: ventricular septal defect.
